# MEMS Hydrophone Signal Denoising and Baseline Drift Removal Algorithm Based on Parameter-Optimized Variational Mode Decomposition and Correlation Coefficient

**DOI:** 10.3390/s19214622

**Published:** 2019-10-24

**Authors:** Huichao Yan, Ting Xu, Peng Wang, Linmei Zhang, Hongping Hu, Yanping Bai

**Affiliations:** 1 School of Information and Communication Engineering, North University of China, Taiyuan 030051, China; b1705012@st.nuc.edu.cn; 2 Department of Mathematics, School of Science, North University of China, Taiyuan 030051, China; xuting@nuc.edu.cn (T.X.); wpmath@nuc.edu.cn (P.W.); s1708029@st.nuc.edu.cn (L.Z.); huhongping@nuc.edu.cn (H.H.)

**Keywords:** whale-optimization algorithm (WOA), variational mode decomposition (VMD), power spectrum entropy (PSE), cross correlation (CC), denoising, baseline drift removal

## Abstract

Underwater acoustic technology is an important means of detecting the ocean. Due to the complex influence of the marine environment, there is a lot of noise and baseline drift in the signals collected by hydrophones. In order to solve this problem, this paper proposes a denoising and baseline drift removal algorithm for MEMS vector hydrophone based on whale-optimized variational mode decomposition (VMD) and correlation coefficient (CC). Firstly, the power spectrum entropy (PSE), which reflects the variation characteristics of the signal frequency is selected as the fitness function of the whale-optimization algorithm to find the parameters (*K*,*α*) of the VMD. It is easier to find the global optimal solution of the parameters by combining the whale-optimization algorithm. Then, using the VMD algorithm after obtaining the parameters, the original signal is decomposed to obtain the intrinsic mode functions (IMFs), and calculating the correlation coefficients (CCs) between the IMFs and the original signal. Finally, the CC threshold is used to remove the noise IMFs, and the rest of the useful IMFs are reconstructed to complete the denoising and baseline drift removal process of the original signals. In the simulation experiments, the algorithm proposed in this paper shows better performance by comparing conventional digital signal-processing methods and the related algorithms proposed recently. Applied in the experiments of a MEMS hydrophone, the effectiveness of the proposed algorithm is also verified. This algorithm can provide new ideas for signal denoising and baseline drift removal.

## 1. Introduction

Most areas of the Earth are covered by the ocean. With the increasing range of human activities, the impact of the marine environment on people’s living environment is increasing. The protection and development of the ocean has also received increasing attention [1,2]. People often use underwater acoustic, optical, electromagnetic and magnetic induction technologies to detect the ocean. Underwater acoustic technology has always been a research hotspot because of its long transmission ranges [3]. A hydrophone is an important tool for collecting underwater acoustic signals. The quality of underwater acoustic signals directly affects the detection accuracy of the seabed environment and target organisms [4,5]. Affected by wind waves, fish schools, and ship navigation, the signals collected by hydrophones inevitably contain baseline drift noise, which brings great difficulties to the extraction, identification and tracking of target signals [6]. Therefore, denoising and baseline drift removal have very important research significance, and they are also the important step for hydrophones to realize their functions.

The marine environment is complex and variable, and underwater acoustic signals often have the characteristics of strong randomness and noises, which creates great difficulties in dealing with underwater acoustic signals using conventional digital signal-processing methods. Fast Fourier transform is widely used because of its small computation, but it is prone to spectrum aliasing when analyzing continuous signals [7]. The filter algorithm selects the appropriate filter function according to the spectral feature distribution of the wanted signal and the noise signal, while the spectral feature distribution of the useful signal and the noise signal in the complex underwater acoustic environment tends to exhibit great randomness, which is difficult to predict in advance [8]. Wavelet analysis is one of the most commonly used methods for processing signals. It is widely used in machinery [9], medical [10], marine technology [11,12] and other fields. Wavelet analysis needs to select the wavelet basis function and decomposition level in advance when processing signals. Processing signals of different characteristics often get different results and are difficult to be widely applied.

By contrast with the wavelet analysis method, Hilbert Huang et al. proposed an adaptive signal processing method, empirical mode decomposition (EMD), for solving non-linear non-stationary time series analysis in 1998, which will perform signal decomposition according to the characteristic time scale of the signal itself. There is no need to set the basis function, but modal aliasing is prone to occur during signal decomposition with EMD [13]; EEMD and CEEMD have improved this defect [14,15]. EEMD eliminates modal aliasing by adding white noise, but there is no guarantee that the white noise introduced can be completely eliminated during the decomposition process. Although CEEMD uses positive and negative relative white noise, the number of intrinsic mode functions (IMF) obtained by each decomposition is different, resulting in randomness of signal decomposition results.

In 2014, Dominique Zosso et al. proposed a completely non-recursive variational mode decomposition model [16]. Compared with EMD and its improved algorithm, variational mode decomposition (VMD) has solid theoretical derivation and stronger robustness for data sampling and noise. However, the key step in the decomposition algorithm is to find the appropriate parameters K and α, where K is the number of intrinsic mode functions, and α is the penalty factor, which affects the decomposition precision of IMFs. Li et al. combined new permutation entropy (NPE) with VMD methods to denoising and feature extraction of ship-radiated noise [17]; [18] achieves denoising of ship noise by performing twice the VMD decompositions on ship noise. However, in these two literatures, the signals are decomposed by VMD to obtain the number of IMFs equal to the number of IMFs whose signals are decomposed by EMD, and α is selected empirically, which makes the above decomposition methods highly subjective. Many scholars have paid attention to the importance of K and α. They use the algorithms such as the grasshopper optimization algorithm [19], fruit fly optimization algorithm [20], permutation entropy [21] and spectral peak search [22] to adaptively search for K or α parameters of VMD. Yan et al. and Wang et al. used agenetic algorithm and multi-objective particle swarm optimization algorithm to find the best K and α in VMD to solve the problem of composite fault of rotating machinery and fault diagnosis of a gearbox [23,24]. However, they only consider the influence of these two parameters on the intrinsic mode function, but ignore the correlation between the intrinsic mode function and the original signal, which may cause the signal to lose some important information in the denoising process. At the same time, the above literature does not pay attention to the solution to the signal baseline drift.

Methods of baseline drift removal are mostly found in the literature on ECG signal processing [25]. The least square fitting and Savitzky-Golay smoothing filter are the most commonly used methods in digital signal processing to remove the baseline drift. However, these two methods have no effect on the denoising of signal details, and further denoising needs to be combined with other methods [26,27]. Verma et al. uses empirical wavelet transform to remove baseline drift noise [28]; Sanyal et al. eliminated baseline wander based on a smooth wavelet tight frame with vanishing moments [29]; Sheetal et al. combines a hybrid derivative and MaMeMi filter to remove baseline drift [30]; Kim et al. semi-real-time removal of baseline fluctuations in ECG signals using an infinite impulse response low-pass filter [31]; These methods require artificial selection of appropriate wavelet basis functions or filter functions, which are subjective and easily lead to signal distortion. In an article presented at the 7th international symposium on sensor science [32], the empirical mode decomposition is used to decompose the signal into intrinsic mode functions and their residual steady-state quantities. The residual steady-state quantity generally reflects the trend term of the signal. Removing this trend term can eliminate the baseline drift, but the effect of this method is generally affected by its decomposition accuracy.

In this paper, we propose a denoising and baseline drift removal algorithm for a MEMS vector hydrophone based on whale-optimized VMD and correlation coefficient. Firstly, the power spectrum entropy which can reflect the signal complexity is selected as the fitness function [33], and the K and α parameters in the VMD are searched for by the whale-optimization algorithm [34]. Then, the original signal is decomposed by the parameter-optimized VMD to obtain IMFs, calculating the correlation coefficients (CCs) of the IMFs and the original signal, and obtaining noise IMFs and useful signal IMFs by setting a threshold. Finally, we reconstruct the useful IMFs to achieve the purpose of denoising and baseline drift removal. In the simulation experiments of signals with different characteristics, compared with conventional digital signal-processing methods and related algorithms proposed recently, the proposed algorithm in this paper has significant effects on signal denoising and baseline drift removal, especially in a strong noise environment, and the signal-to-noise ratio is increased by more than 90%. At the same time, this paper analyzes the advantages of a whale-optimization algorithm (WOA) in finding VMD parameters and the principle of baseline drift removal. Applied in the experiments of MEMS hydrophone, the proposed algorithm achieves good effectiveness in denoising and baseline drift removal.

The rest of the article is organized as follows: in the second part, the theoretical basis of VMD, whale-optimization algorithm, power spectrum entropy and correlation coefficient are introduced. The specific steps of the proposed algorithm are introduced in the third part. In the fourth part, the proposed algorithm was simulated and compared with other algorithms. The application in MEMS hydrophones of the proposed algorithm and the conclusions are in Parts 5 and Parts 6, respectively.

## 2. Theoretical Basis

### 2.1. Variational Mode Decomposition (VMD)

VMD is a completely non-recursive variational model decomposition model whose purpose is to decompose the original signal into intrinsic mode functions (IMFs) with fixed bandwidth and center frequency. The decomposition process of the original signal is actually the solution process of constructing the variational function problem.

The definition of the intrinsic mode function is as follows:

(1) $$u_{k}(t)=A_{k}(t)\cos(\phi_{k}(t))$$

where $u_{k}(t)$ represents the intrinsic mode function (IMF), $A_{k}(t)$ represents the envelope of the IMF; $\phi_{k}(t)$ represents the phase, which is a non-attenuating function, and t represents time.

Then, the bandwidth of the IMF is estimated by demodulation, and the constraint variation model is given as follows:

(2) $$\begin{array}{c} \underset{\left\{u_{k}\},\{\omega_{k}\right\}}{\min}\left\{\sum_{k}{\left\|\partial_{t}\left[\left(\delta (t)+\frac{j}{\pi t}\right)*u_{k}(t)\right]e^{-j\omega_{k}t}\right\|}_{2}^{2}\right\}\\ s.t.\sum_{k}u_{k}=x(t) \end{array}$$

where $\left\{u_{k}\}=\{u_{1},\cdots ,u_{K}\right\}$ and $\left\{\omega_{k}\}=\{\omega_{1},\cdots ,\omega_{K}\right\}$ represent IMFs and their center frequencies, respectively; *K* represents the number of IMFs, x(t) represents the original signal.

In order to solve the optimal solution problem of (2), the algorithm introduces Lagrangian multiplier λ and penalty factor α. The augmented Lagrangian function is defined as follows:

(3) $$L\left(\{u_{k}\},\{\omega_{k}\},\lambda \right)=\alpha {\sum_{k}\left\|\partial_{t}\left[\left(\delta (t)+\frac{j}{\pi t}\right)*u_{k}(t)\right]e^{-j\omega_{k}t}\right\|}_{2}^{2}+{\left\|f\left(t\right)-\sum_{k}u_{k}\left(t\right)\right\|}_{2}^{2}+\langle \lambda \left(t\right),f\left(t\right)-\sum_{k}u_{k}\left(t\right)\rangle$$

Finally, the optimal solution of the constrained variational model is solved by the alternating direction multiplier method (ADMM), and $u_{k}$, $\omega_{k}$ and λ are updated in the frequency domain. The update formula is as follows:

(4) $${\hat{u}}_{k}^{n+1}(\omega )=\frac{\hat{f}(\omega )-\sum_{i<k}{\hat{u}}_{i}^{n+1}(\omega )-\sum_{i>k}{\hat{u}}_{i}^{n}(\omega )+\frac{{\hat{\lambda }}^{n}(\omega )}{2}}{1+2\alpha {\left(\omega -\omega_{k}^{n}\right)}^{2}}$$

(5) $$\omega_{k}^{n+1}=\frac{\int_{0}^{\infty }\omega {\left|{\hat{u}}_{k}^{n+1}(\omega )\right|}^{2}d\omega }{\int_{0}^{\infty }{\left|{\hat{u}}_{k}^{n+1}(\omega )\right|}^{2}d\omega }$$

(6) $${\hat{\lambda }}^{n+1}(\omega )={\hat{\lambda }}^{n}(\omega )+\tau \left(\hat{f}(\omega )-\sum_{k}{\hat{u}}_{k}^{n+1}(\omega )\right)$$

the termination conditions are as follows:

(7) $${\sum_{k}{\left\|{\hat{u}}_{k}^{n+1}-{\hat{u}}_{k}^{n}\right\|}_{2}^{2}/\left\|{\hat{u}}_{k}^{n}\right\|}_{2}^{2}<\varepsilon$$

where ε represents convergence accuracy, and the specific steps of the VMD algorithm are as follows:

Step 1: Initialize the parameters, set $\left\{{\hat{u}}_{k}^{1}\right\}$, $\left\{\omega_{k}^{1}\right\}$, ${\hat{\lambda }}^{1}$ and n to 0;
Step 2: Update the values of $\left\{{\hat{u}}_{k}^{n+1}\right\}$, $\left\{\omega_{k}^{n+1}\right\}$, and ${\hat{\lambda }}^{n+1}$ according to Formulas (4)–(6);
Step 3: Determine whether the termination condition (7) is satisfied, and repeat step 2 until Formula (7) is satisfied.

### 2.2. Whale-Optimization Algorithm (WOA)

In 2016, Mirjalili and Lewis proposed a new meta-heuristic optimization algorithm for simulating humpback whale hunting behavior called the whale-optimization algorithm (WOA). The algorithm is inspired by the humpback whale’s bubble net foraging behavior [35]. The humpback whale first creates a large number of spiral bubbles around the prey to surround them and then hunted. During the predation process, humpback whales have two predation strategies, one is to reduce the surrounding mechanism, and the other is the spiral update position strategy. In each predation, these two strategies are used simultaneously. To simulate these two strategies, the whale-optimization algorithm introduces a random selection probability p(p∈[0,1]). When p≥0.5, the humpback whale uses the spiral update position strategy to prey; when p<0.5, the humpback whales prey on the strategy of the shrinking encircling mechanism. In the strategy of the shrinking encircling mechanism, the whales randomly search for the prey according to the position of each other. In order to reflect this randomness in the algorithm, the algorithm introduces the coefficient vector $\vec{A}$. A random search agent is chosen when $\left|\vec{A}\right|>1$, while the best solution is selected when $\left|\vec{A}\right|<1$ for updating the position of the search agents. Because it has multiple search strategies, it is easier to find the global optimal solution, which has a strong advantage compared with the traditional optimization algorithm [36,37]. The important mathematical model of the algorithm is as follows:

(8) $$\vec{D}=\left|\vec{C}.\vec{X^{*}}(t)-\vec{X}(t)\right|$$

where t represents the current number of iterations, $\vec{X}$, $X^{*}$ represent the position vector and the position vector of the best solution obtained so far respectively; $\vec{C}$ is the coefficient vector, and $\vec{D}$ is the search distance.

The definition of a random search agent:

(9) $$\vec{X}(t+1)=\vec{X_{rand}}-\vec{A}.\vec{D}$$

where $\vec{X_{rand}}$ is the random position vector (a random whale) selected from the current population, $\vec{A}$ is the coefficient vector.

When the humpback whale uses the spiral updating position strategy to prey, its spiral equation is:

(10) $$\vec{X}(t+1)=\vec{D^{\prime }}\cdot e^{bl}\cdot \cos(2\pi l)+\vec{X^{*}}(t)$$

where $\vec{D^{\prime }}=\left|\vec{X^{*}}(t)-\vec{X}(t)\right|$ indicates the distance of the *i*-th whale to the prey (best solution obtained so far; b is a constant for defining the shape of the logarithmic spiral; l is a random number in [−1,1]; ⋅ is the multiplication of elements with elements.

**Figure 1:**
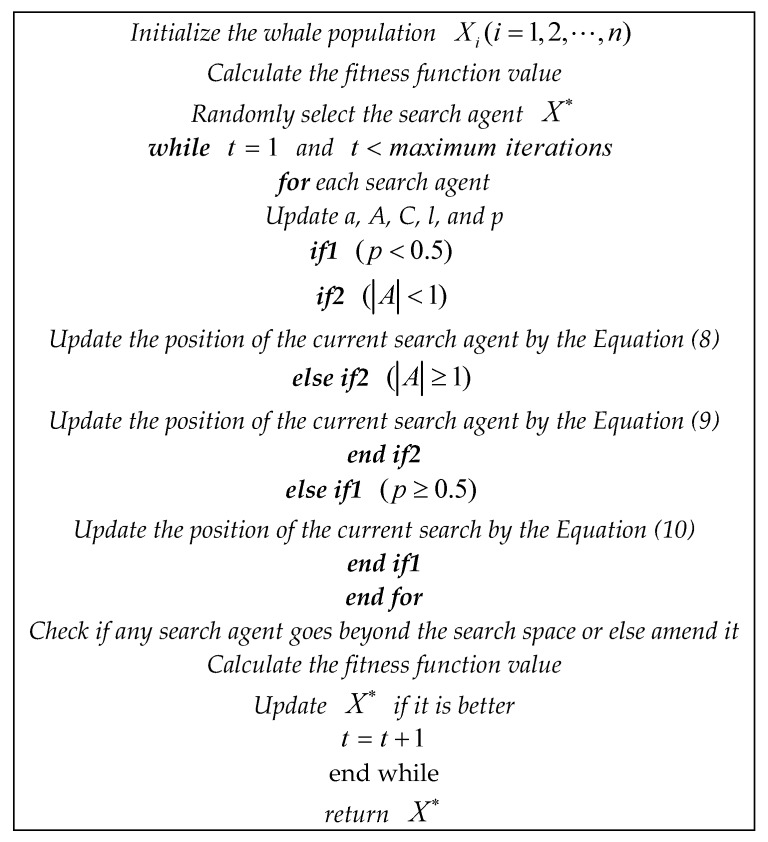
Pseudo code of the whale-optimization algorithm (WOA) algorithm.

The pseudo code of the whale-optimization algorithm is shown in Figure 1:

### 2.3. Power Spectrum Entropy (PSE)

Shannon, C. E. proposed the concept of information entropy in 1948, providing a theoretical basis for the quantitative description of uncertain information [38]. The power spectrum entropy (PSE) is an extension of the information entropy in the frequency domain, which can be used to describe the variation characteristics of the frequency. It can reflect the complexity of the signal frequency composition in chatter detection and gearbox fault diagnosis [24,33]. In order to reflect the complexity of each frequency component of the underwater acoustic signal, the power spectral entropy is selected as the fitness function of the whale-optimization algorithm, and the smaller the power spectral entropy, the stronger the signal sparsity, the more it can reflect the frequency distribution of the underwater acoustic signal.

The algorithm steps of power spectrum entropy are as follows:

Step 1: Calculation formula of the power spectrum of signal x(t):
  (11) $$s(f)=\frac{1}{2\pi L}{\left|x(w)\right|}^{2}$$
  where L is the signal length and x(w) is the Fourier transform of x(t);
Step 2: Obtain the probability density function of the spectrum of all frequency components by normalization:
  (12) $$P_{i}=\frac{s(f_{i})}{\sum_{k=1}^{N}s(f_{k})}\left(i=1,2,3,\cdots ,N\right)$$
  where $s(f_{i})$ is the spectral energy of frequency component $f_{i}$; $P_{i}$ is the corresponding probability density; N is the number of frequency components in the fast Fourier transform of the total probability density;
Step 3: The PSE value is defined as:
  (13) $$H=-\sum_{k=1}^{N}P_{i}\log P_{i}$$

### 2.4. Correlation Coefficient (CC)

In order to remove the noise component after signal decomposition by VMD and maintain the correlation between the denoised signal and the original signal, a correlation coefficient function is introduced in this paper. The correlation coefficient makes the denoised signal contain the main features of the original signal. The definition of correlation coefficient is as follows:

(14) $$R=\frac{E\left[u_{i}(t).x(t)\right]-E\left[u_{i}(t)\right]E\left[x(t)\right]}{\sqrt{D\left[u_{i}(t)\right]}\sqrt{D\left[x(t)\right]}}$$

where R represents the number of CCs between the IMFs and the original signal, x(t) and $u_{i}(t)$ represent the original signal and the IMF, respectively, and *E* and *D* represent the expectation and variance in mathematics.

## 3. The Method Proposed in This Paper (WOA–VMD–CC)

Combining the above analysis and theoretical basis, a denoising and baseline drift removal algorithm for MEMS vector hydrophone based on whale-optimization VMD and correlation coefficient is proposed. The algorithm steps are as follows:

Step1: Aiming at the input signal, the whale-optimization algorithm (WOA) with the fitness function PSE is used to find the optimal parameters K (the number of intrinsic mode functions) and α (penalty factor) of the VMD algorithm; In this paper, the (K,α) parameter pair is used as the position vector for the whale population in the WOA algorithm. According to the characteristics of general ocean noise, different types of noises show different frequency distribution characteristics, showing strong sparsity, so when the value of the PSE of each IMF is smaller, the effect obtained by the decomposition of the underwater acoustic signal is better. The process by which WOA optimizes the parameters (K,α) of the VMD is finding a position vector that minimizes the power spectral entropy. This article sets K∈[3,9],α∈[1000,9000],K,α∈Z. At the same time, in order to make the WOA faster and more accurate to find the optimal parameter pair, this paper takes the population number $X_{i}=10$ and the maximum number of iterations mI=50.

Step2: Using the optimal parameters *K* and *α* obtained in the first step to decompose the original signal by VMD and obtain IMFs.

Step 3: Calculate the CCs between each IMF and the original signal. In this paper, [18] set the threshold of CC to 0.2, the IMF, whose CC is smaller than the threshold is the noise IMF, and the IMF with CC greater than or equal to the threshold is the useful IMF;

Step 4: Reconstruct the useful IMFs to obtain the result of denoising and baseline drift removal of the original signal.

**Figure 2:**
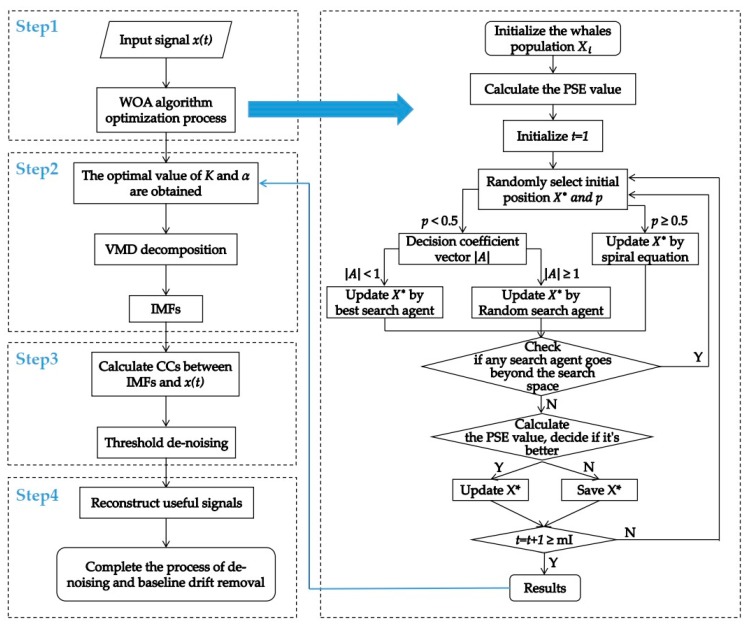
The algorithm proposed in this paper.

The algorithm flow proposed in this paper is shown in Figure 2.

## 4. Simulation

In order to verify the effectiveness and advantages of the proposed algorithm in denoising and baseline drift removal of underwater acoustic signals, through the analysis of the characteristics of underwater acoustic signal noise, this part will simulate the proposed algorithm through two sets of analog signals, and compared with conventional digital signal-processing methods and the related algorithm proposed in recent years. At the same time, in the simulation experiment 1, this paper analyzes the importance of the parameter (K,α) selection of VMD and carries out experimental verification. In comparison with the related algorithms proposed recently, the comparison algorithm 1 is a ship noise denoising method (VMD–NPE) combining VMD and NPE [17]; and comparison algorithm 2 is a ship noise denoising algorithm combined with secondary variational mode decomposition and correlation coefficients (2VMD-CC) [18]. The parameters *K* and *α* of the VMD in these two algorithms are given by the number of IMFs of EMD and empirical values, respectively. Comparison algorithm 3 is a hydroacoustic signal denoising algorithm combining empirical mode decomposition and wavelet analysis (EMD-WT) [32]. In comparison with conventional digital signal-processing methods, comparison algorithm 4 combines least squares fitting with wavelet soft threshold to process the signal (LSF–WST), and comparison algorithm 5 combines the Savitzky–Golay smoothing filter with wavelet soft threshold to process the signal (SGSF–WST).

### 4.1. Simulation Experiment 1

**Figure 3:**
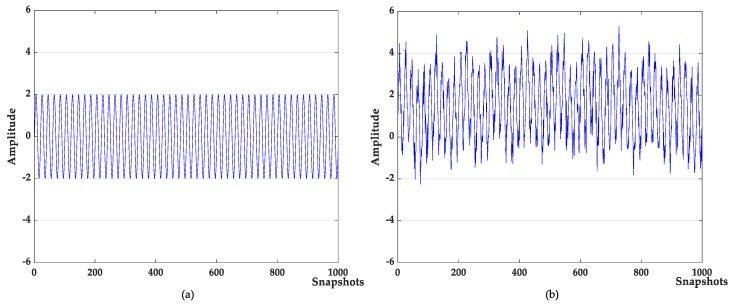
(**a**) target signal; (**b**) simulation signal.

**Figure 4:**
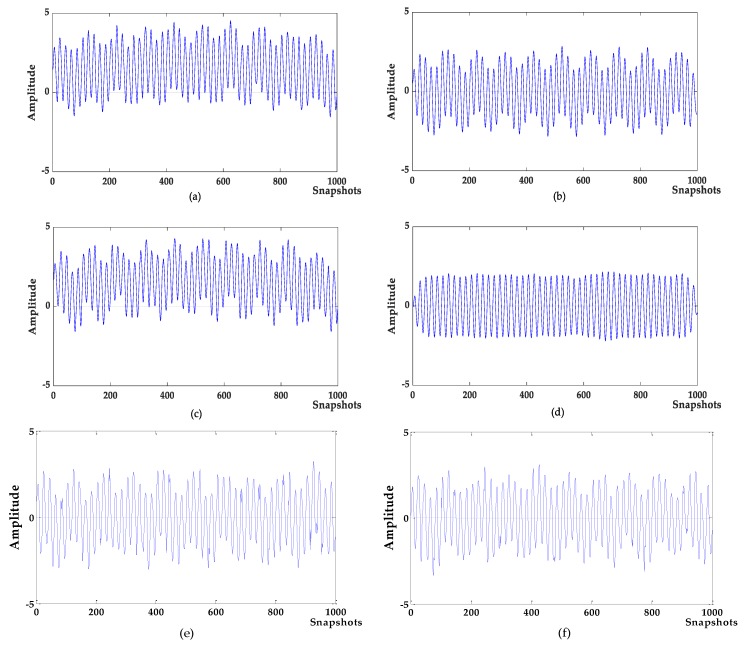
The denoising results of different algorithms. (**a**) variational mode decomposition-new permutation entropy (VMD-NPE); (**b**) 2VMD-correlation coefficient (CC); (**c**) empirical mode decomposition and wavelet analysis (EMD-WT); (**d**) WOA-VMD-CC; (**e**) least squares fitting with wavelet soft threshold (LSF-WST); (**f**) Savitzky–Golay smoothing filter (SGFT)-WST.

Robert Urick et al. analyzed the noise of the marine environment and found that the source of the main noise is different in different sound frequency bands [39]. Therefore, it is necessary to analyze the denoising and baseline drift removal of mixed signals with different frequencies of noises. In the underwater acoustic mixed signal, the signal to be detected is the target signal, and the other signals are noise signals. To this end, in this section, the simulation signal S consists of the target signal f and other frequency signals $f_{1},f_{2},f_{3}$ and the baseline drift signal $f_{4}$ and 0.5 times the standard Gaussian white noise n, as shown in Equation (15):

(15) $$\left\{ \begin{array}{l} f=2\sin(2\pi 500t)\\ f_{1}=0.8\sin(2\pi 5t)\\ f_{2}=0.6\sin(2\pi 100t)\\ f_{3}=0.3\sin(2\pi 2000t)\\ f_{4}=1\\ n=0.5randn(t)\\ S=f+f_{1}+f_{2}+f_{3}+f_{4}+n \end{array}\right.$$

where f is the target signal with a frequency of 500 Hz, and $f_{1},f_{2},f_{3}$ are the signals with frequency of 5 Hz, 100 Hz, and 2000 Hz, respectively; $f_{4}$ is the baseline drift signal and the sampling frequency is 1000 Hz. The waveforms of the target signal and the simulated signal are shown in Figure 3. The simulated signal contains obvious noises and baseline drift. The denoising results of different algorithms are shown in Figure 4. The denoising evaluation indexes of different algorithms are recorded in Table 1.

Denoising analysis: It can be seen from Figure 3 and Figure 4 that these algorithms have denoising effects on the simulated signals. The denoising results of VMD-NPE, 2VMD-CC and EMD-WT still have some amplitude oscillation, indicate that the components of their denoised signals are mixed. The waveforms denoised by LSF-WST and SGSF-WST are not only undulating, but also have many burr noises in the upper and lower parts. The amplitude of the signal denoised by the algorithm proposed in this paper is more stable, and its waveform is closer to the target signal. It can be seen from Table 1 that in SNR and root mean squared error (RMSE), the proposed algorithm is more advantageous than other algorithms, especially in terms of signal-to-noise ratio.

Baseline drift removal effect analysis: the algorithm in Figure 4a,c does not remove the baseline drift of the signal, and the 2VMD-CC, LSF-WST, SGSF-WST algorithms overall waveforms are in the middle position, but there are still waveform fluctuations; The waveform of the algorithm proposed in this paper is in the zero level, and there is no large fluctuation, which is closer to the target signal, and the effect of baseline drift removal is obvious.

**Figure 5:**
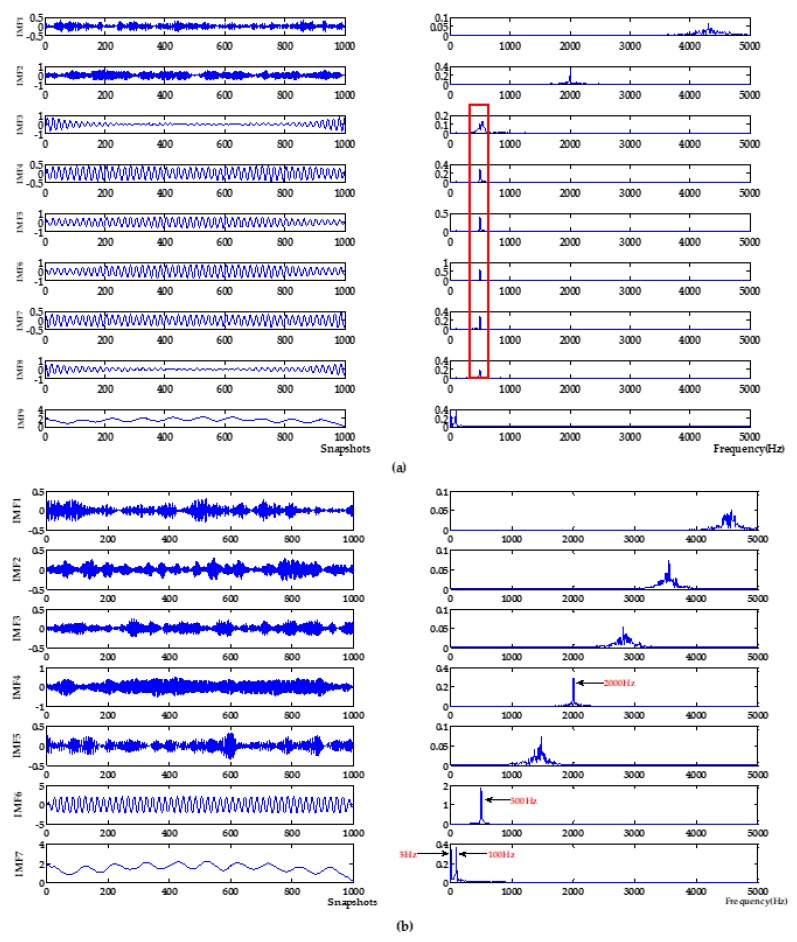
VMD time-frequency diagram with different parameters. (**a**) *K* is equal to the decomposition level by EMD, α is the empirical value of 2000. (**b**) Parameters obtained by WOA, *K* is 7, α is 6240.

**Figure 6:**
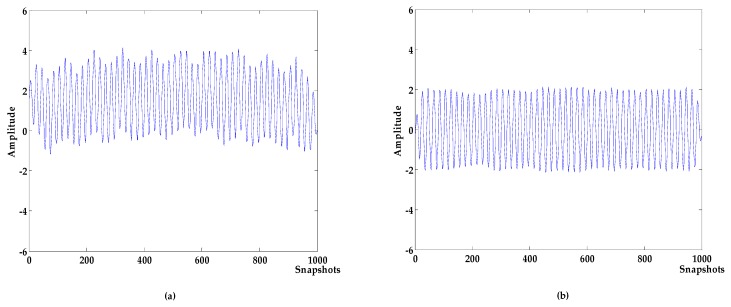
The denoising results of WOA-VMD-NPE and WOA-2VMD-CC. (**a**) WOA-VMD-NPE; (**b**) WOA-2VMD-CC.

In addition, VMD-NPE, 2VMD-CC and the proposed algorithm decompose the original signal through VMD firstly, and then perform denoising and baseline drift removal processing. Among them, the VMD-NPE algorithm is different from the other two algorithms in further processing, resulting in poor denoising and baseline drift removal; the 2VMD-CC algorithm and the algorithm proposed in this paper both use the threshold of CCs for further processing. Although 2VMD-CC has certain effect on baseline drift removal, the algorithm proposed in this paper has obvious advantages in denoising effect. It can be seen from Table 1 that the signal-to-noise ratio of the algorithm in this paper is much higher than the signal-to-noise ratio of 2VMD-CC. This shows that the selection of VMD parameters (K,α) and further processing are very important for signal denoising and baseline drift removal. In order to illustrate the importance and advantages of the WOA algorithm in the process of searching VMD parameters in this paper, the decomposition process of VMD with different parameters was analyzed. The parameters of VMD in VMD-NPE and 2VMD-CC were selected using the parameter-searching method of WOA combined with PSE proposed in this paper, which are defined as WOA-VMD-NPE and WOA-2VMD-CC algorithms, respectively, and simulation experiments were carried out. VMD time-frequency diagrams with different parameters are shown in Figure 5. The denoising results of the WOA-VMD-NPE and WOA-2VMD-CC are shown in Figure 6. The denoising evaluation indexes of VMD-NPE, WOA-VMD-NPE, 2VMD-CC, WOA-2VMD-CC, and algorithm proposed in this paper are recorded in Table 2.

It can be seen from Figure 5 that the modal aliasing occurs when the parameter *K* of VMD is equal to the number of IMFs which are obtained by decomposing signals using EMD, and the parameter *α* is selected empirically. The VMD of the selected parameters of the algorithm in this paper decomposes the original signal into multiple frequency bands without aliasing. According to the simulation signal Formula (15), it can be seen that signals of multiple frequencies are decomposed, with a single frequency component and better decomposition effect. It can be seen from Figure 6 and Table 2 that the WOA-VMD-NPE makes some improvement to the denoising effect compared with the VMD-NPE, but the baseline drift still exists. Compared with 2VMD-CC, WOA-2VMD-CC makes a significant improvement in denoising and baseline drift removal, but the algorithm proposed in this paper is better on SNR and RMSE. It shows that the WOA-2VMD-CC has excessive denoising in the denoising process, and it is more cumbersome in the algorithm steps. The process of removing the baseline drift is actually the process of removing the baseline drift IMF, so whether the baseline drift component can be decomposed and removed effectively is the key to remove the baseline drift of the signal. The above analysis shows that using WOA to search the parameters of VMD can effectively improve the accuracy of VMD algorithm, and it is very important to improve performance for the signal denoising and baseline drift removal.

### 4.2. Simulation Experiment 2

**Figure 7:**
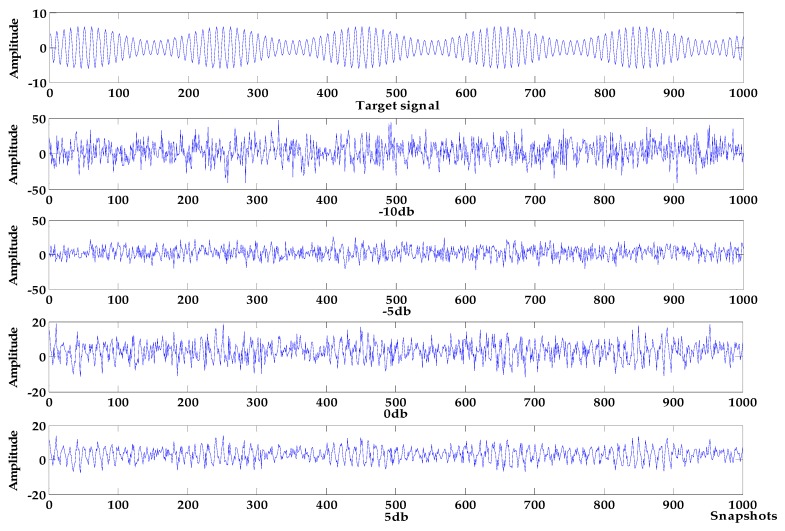
Target signal and simulated signal.

**Figure 8:**
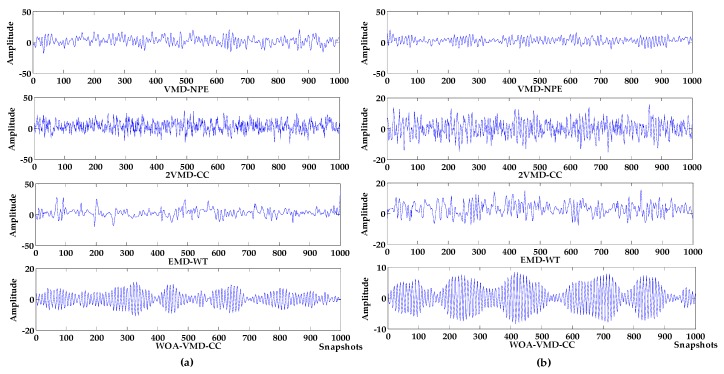

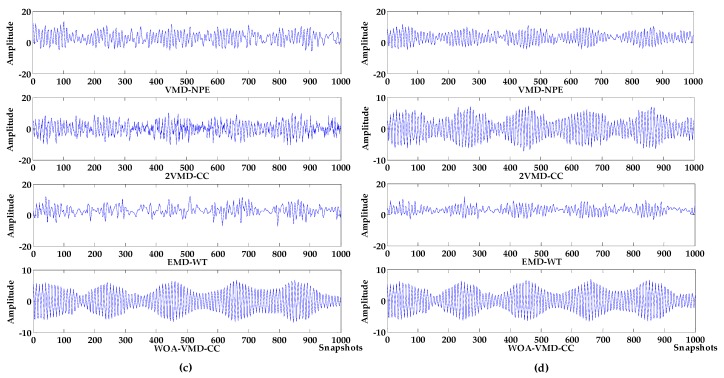
Denoising results of different algorithms under different decibel noises. (**a**) −10 db noise; (**b**) −5 db noise; (**c**) 0 db noise; (**d**) 5 db noise.

**Figure 9:**
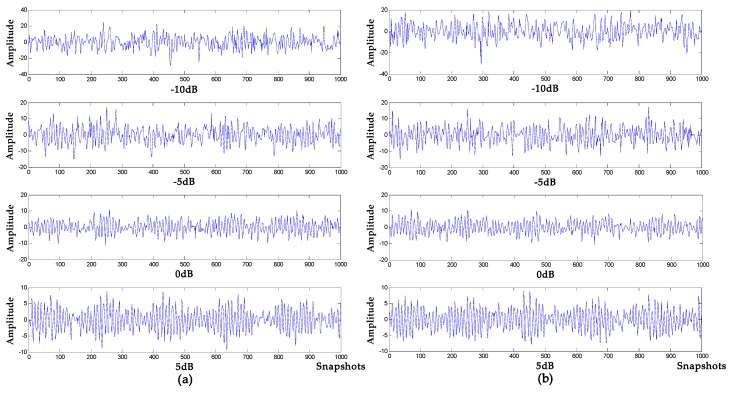
Denoising and baseline drift removal results of traditional digital signal processing methods in different noise intensity environments. (**a**) LSF-WST; (**b**) SGSF-WST.

Due to the influence of the marine environment and human activities, the noise intensity of the underwater acoustic signal is variable. To simulate this situation, the simulated signal S consists of the target signal f and the baseline drift signal $f_{1}$ and Gaussian white noise of different decibels, as shown in (16):

(16) $$\left\{ \begin{array}{l} f=2\cos(2\pi 100t)(sin(2\pi 5t)+2)\\ f_{1}=3\\ n=white\;noise\;at\;different\;decibels\\ S=f+f_{1}+n \end{array}\right.$$

where the target signal f is composed of an amplitude modulation signal, and the noise signals are Gaussian white noises of −10 db, −5 db, 0 db and 5 db, respectively, and the sampling frequency is 1000 Hz. Waveform diagrams of the target signal and the simulated signals are shown in Figure 7, The denoising results of VMD-NPE, 2VMD-CC, EMD-WT and WOA-VMD-CC are shown in Figure 8; Figure 9 shows the denoising results of LSF-WST and SGSF-WST; and the denoising evaluation indicators of various algorithms are recorded in Table 3.

It can be seen from Figure 7, Figure 8 and Figure 9 that in the environment where the noise decibel is −10 db, −5 db, 0 db, the denoising results of VMD-NPE, 2VMD-CC, EMD-WT, LSF-WAT and SGSF-WST are all distorted. From the trend of signal change, the signal denoised by the algorithm proposed in this paper reflects the characteristics of the target signal change better. In the 5 db noise environment, the signals denoised by VMD-NPE, EMD-WT, LSF-WAT and SGSF-WST are still distorted. 2VMD-CC has a certain denoising effect on the simulated signal, but there are many high-frequency noises in the upper and lower parts of the denoised signal. The denoised signal waveform of the algorithm proposed in this paper is smoother, and the denoising effect is better. It can be seen from Table 3 that the denoising performance of the algorithm proposed in this paper under different decibel noises is better than the other algorithms. In terms of baseline drift removal, it can be seen from Figure 8 that neither the VMD-NPE nor the EMD-WT algorithm solves the baseline drift problem. The stronger the noise, the worse the 2VMD-CC baseline drift removal effect. In the −10db noise environment, 2VMD-CC does not remove the baseline drift; The algorithm proposed in this paper removes the baseline drift in the different simulation experiments above. In Figure 9, the LSF-WAT and SGSF-WST algorithms remove baseline drift, but the signal denoising effect is poor. In addition, when removing baseline drift, conventional digital signal methods usually fit the trend term from the low-frequency component of the signal, and then remove the trend term from the original signal to achieve the purpose of removing baseline drift. In the ocean, the useful underwater acoustic signal usually has the characteristics of low frequency, and it is easy to lose the useful information by using the conventional digital signal-processing method. By decomposing the underwater acoustic signal accurately, the denoising and baseline drift removal process retains more useful information. In order to verify the above reasoning, this paper carried out the following experiment. The simulation signal is shown in Equation (16), and the Gaussian white noise is 5 db. First, the simulated signal removes baseline drift by least squares fitting and Savitzky–Golay smoothing filter respectively. Then, we use the algorithm proposed in this paper for denoising. The verification algorithms are respectively recorded as LSF-WOA-VMD-CC and SGSF-WOA-VMD-CC. Different denoising evaluation indexes of different algorithms are recorded in Table 4.

As can be seen from Table 4, the proposed algorithm in this paper is better than LSF-WOA-VMD-CC and SGSF-WOA-VMD-CC. Combined with the above simulation results, the proposed algorithm is better than the other algorithm in denoising and baseline drift removal.

## 5. Application in the Experiments of MEMS Hydrophone

In order to verify the application effect of the proposed algorithm in practical work, it is applied to the experiments of hydroacoustic signal processing of MEMS vector hydrophone.

### 5.1. MEMS Vector Hydrophone and Signal Acquisition

**Figure 10:**
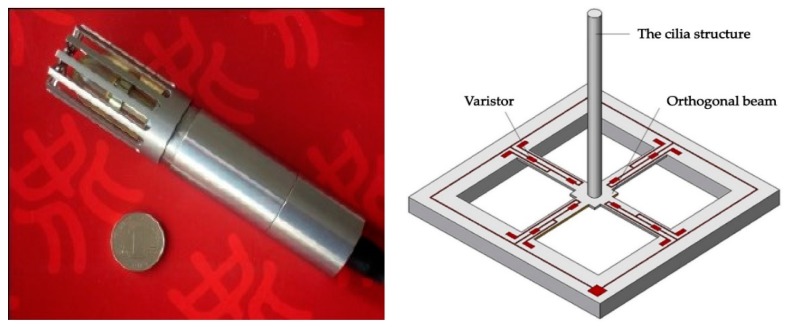
The basic structure of the hydrophone.

The hydrophone used in the experiments is a MEMS vector hydrophone produced by North University of China [40]. The basic structure of the hydrophone is shown in Figure 10. The cilia vibration caused by sound wave propagation produces the stress, which deforms the root of the cross beam micro-structure. The resistance of the varistor integrated at the root of the cross beam is changed. Then the resistance change of *X*-axis, *Y*-axis are transferred to voltages, and measured by Wheatstone bridge. Following the principle, the underwater sound signal is converted into the electrical signal.

**Figure 11:**
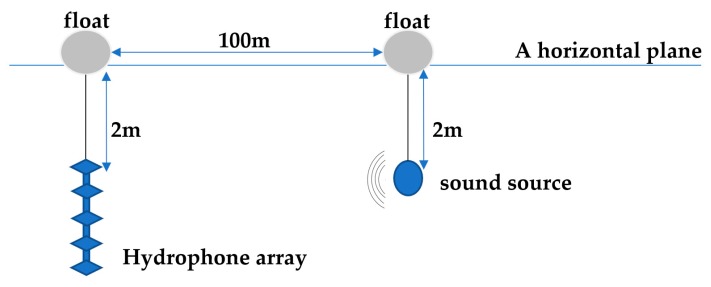
The procedure of collecting the underwater acoustic signal.

The procedure of collecting the underwater acoustic signal is shown in Figure 11. At two meters above the water level, the hydrophone array consists of five vertically distributed hydrophones, named from the top to the bottom of the hydrophone 1–5. The sound source is located at a distance of 100 m. Each experiment transmits a signal of a frequency, and the hydrophone is sampled at a frequency of 10 kHz.

### 5.2. Denoising and Baseline Drift Removal Experiments for MEMS Vector Hydrophones

**Figure 12:**
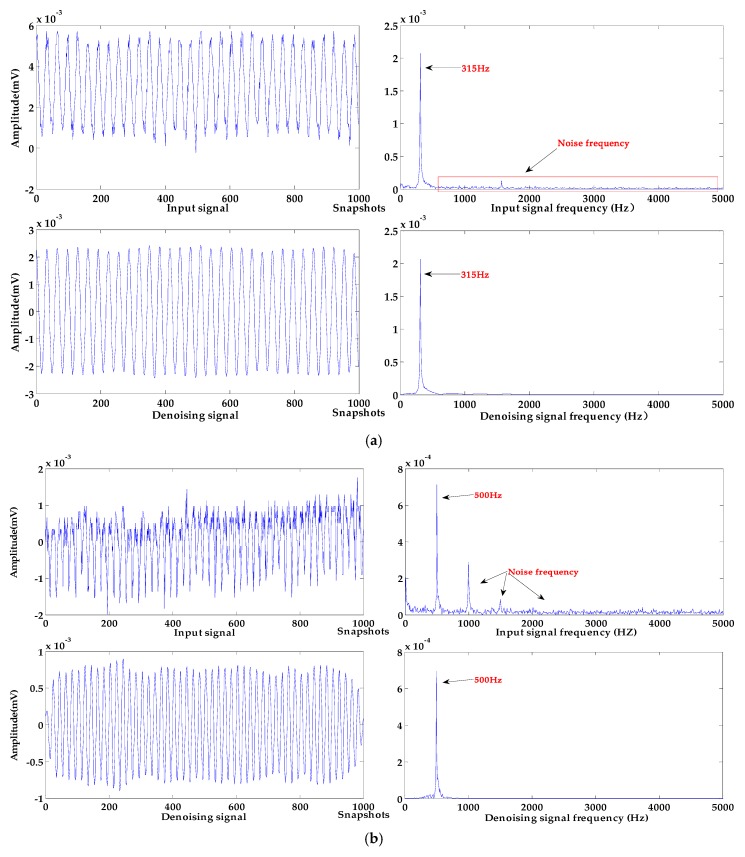

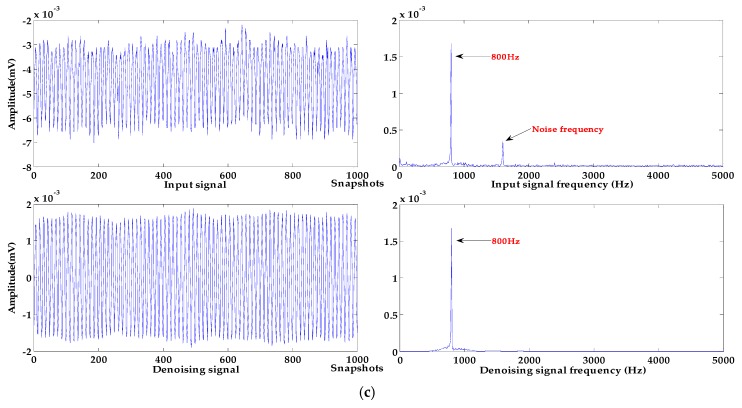
Processing results of different measured signals. (**a**) sound source frequency 315 Hz; (**b**) sound source frequency 500 Hz; (**c**) sound source frequency 800 Hz.

The signals of different frequencies with the baseline drift that occurred in the experiment were selected, and the algorithm proposed in this paper was used for denoising and baseline drift removal. The signal with 1000 sampling points were randomly selected for the denoising and baseline drift removal experiments in this work. The processing results of signals of different frequencies are shown in Figure 12.

As can be seen from Figure 12a, the frequency of the input signal is 315 Hz, and it has an upward baseline drift and contains a few high frequency noises. In Figure 12b, the input signal at 500 Hz has a baseline drift to the upper right, a lot of strong high-frequency noises cause serious distortion of the signal. In Figure 12c, the frequency of the input signal is 800 Hz, and the baseline of the input signal is largely deviated from the zero level; high-frequency noise causes irregular changes in the amplitude of the input signal.

These input signals are processed by the algorithm proposed in this paper; the baseline of each denoised signal has been effectively corrected to the zero level, and the baseline drift for different trends have been removed. In terms of denoising effect, the noise of all input signals is effectively removed. The input signal with a frequency of 315 Hz has a few high-frequency noises, the signal waveform after denoising is smoother, and the denoising effect is the best. After denoising the input signals with frequencies of 500 Hz and 800 Hz, the amplitude of the denoising signal is stable, which retains the basic characteristics of the sound source signal. It can be seen from the spectrograms that the high-frequency noises are effectively removed and the energy of denoised signals are not reduced.

In summary, in the application experiments of a MEMS hydrophone, the algorithm proposed in this paper can effectively denoise and remove baseline drift of hydroacoustic signals in different frequency ranges and noise intensity. It can provide ideas for denoising and baseline drift removal of underwater acoustic signals, and can also provide technical support for the application of hydrophones in practical engineering.

## 6. Conclusions

In order to solve the problem of signal distortion and baseline drift caused by noise, this paper proposes a denoising and baseline drift removal algorithm for a MEMS vector hydrophone based on whale-optimized VMD and CC. Through a lot of experiments and analysis, the proposed algorithm demonstrates a good effect in denoising and baseline drift removal. The superiority and contribution of the algorithm are as follows:

(1) In many literatures, the parameters of VMD are selected by empirical method. In this paper, the whale-optimization algorithm is used to optimize the parameters (K,α) of the VMD. While taking into account the mutual influence between the two parameters, it is easier to find the global optimal solution, which provides an idea for adaptively searching for the parameters of the VMD.
(2) Power spectrum entropy (PSE) can reflect the variation characteristics of the frequency in the signal. In the whale-optimization algorithm, PSE is used as the fitness function, and it is easier to find the optimal parameters (K,α), which can improve the accuracy of signal decomposition. There is no modal aliasing when decomposing the signal using the algorithm proposed in this paper.
(3) This paper calculates the CCs of the IMFs and the original signal, and denoises the signal by setting the CC threshold. The correlation between the denoised signal and the original signal is fully considered, so that the denoised signal retains more important information of the original signal.
(4) Conventional digital signal-processing methods tend to lose useful information when removing baseline drift. The algorithm in this paper has a good performance for baseline drift removal of signals with different characteristics. At the same time, more useful information is retained.
(5) Compared with conventional digital signal-processing methods and other related algorithms proposed recently, the denoising effect of this algorithm is better.

## Figures and Tables

**Table 1:** Denoising evaluation indexes of different algorithms.

Index	Input Signal	VMD-NPE	2VMD-CC	EMD-WT	WOA-VMD-CC	LSF-WST	SGSF-WST
SNR	5.8810	9.3281	9.4184	7.8212	**18.5693**	8.5445	8.5935
RMSE	1.6560	1.5796	0.4782	1.6382	**0.1667**	0.5288	0.5030

**Table 2:** Denoising evaluation indexes of VMD-NPE, WOA-VMD-NPE, 2VMD-CC, WOA-2VMD-CC and WOA-VMD-CC.

Index	VMD-NPE	WOA-VMD-NPE	2VMD-CC	WOA-2VMD-CC	WOA-VMD-CC
SNR	9.3281	10.7579	9.4184	17.0467	**18.5693**
RMSE	1.5796	1.5480	0.4782	0.1987	**0.1667**

**Table 3:** Different denoising evaluation indexes of different algorithms under different decibel noises.

Noise	Index	Input Signal	VMD-NPE	2VMD-CC	EMD-WT	WOA-VMD-CC	LSF-WST	SGSF-WST
−10 db	SNR	−12.9540	−5.4163	−8.3212	−6.1719	**1.3264**	−7.1079	−7.9754
	RMSE	13.6503	6.0592	8.3504	6.7044	**2.5752**	6.8001	7.5148
−5 db	SNR	−7.9970	−1.6381	−2.5925	−1.9593	**5.8786**	−1.4992	−2.6494
	RMSE	8.0206	4.5489	4.0434	4.7587	**1.5247**	3.5635	4.0707
0 db	SNR	−2.9637	3.2030	1.9962	1.6200	**12.3969**	2.0547	2.0923
	RMSE	5.1673	3.6328	2.3840	3.8343	**0.7199**	2.3680	2.3585
5 db	SNR	2.0469	8.7231	11.0416	4.9440	**15.1702**	6.1727	6.4195
	RMSE	3.8618	3.2409	0.8415	3.4430	**0.5231**	1.4740	1.4328

**Table 4:** Different denoising evaluation indexes of LSF-WOA-VMD-CC, SGSF-WOA-VMD-CC and WOA-VMD-CC.

Noise	Index	Input Signal	LSF-WOA-VMD-CC	SGSF-WOA-VMD-CC	WOA-VMD-CC
5db	SNR	2.0469	11.5326	13.3392	**15.1702**
	RMSE	3.8618	0.7952	0.6459	**0.5231**

## References

[1] Halpern B.S., Frazier M., Afflerbach J., Lowndes J.S., Micheli F., O’Hara C., Scarborough C., Selkoe K.A. (2019). Recent pace of change in human impact on the world’s ocean. Sci. Rep..

[2] Principe P.P., Fisher W.S. (2018). Spatial Distribution of Collections Yielding Marine Natural Products. J. Nat. Prod..

[3] Li Y., Wang S., Jin C., Zhang Y., Jiang T. (2019). A Survey of Underwater Magnetic Induction Communications: Fundamental Issues, Recent Advances, and Challenges. IEEE Commun. Surv. Tutor..

[4] Flandrin P., Gonçalvès P., Rilling G. (2004). Detrending and denoising with empirical mode decompositions. Proceedings of the 12th European Signal Processing Conference (EUSIPCO’04).

[5] Tu C.-K., Jiang Y.-Y. (2005). Development of noise reduction algorithm for underwater signals. Proceedings of the 2004 International Symposium on Underwater Technology (IEEE Cat. No.04EX869).

[6] Baskar V.V., Rajendran V., Logashanmugam E. (2015). Study of different denoising methods for underwater acoustic signal. J. Mar. Sci. Technol..

[7] Zhang X., Zhou R., Zhang W. (2019). Improved local cepstrum and its applications for gearbox and rolling bearing fault detection. Meas. Sci. Technol..

[8] Bahaz M., Benzid R. (2018). Efficient algorithm for baseline wander and powerline noise removal from ECG signals based on discrete Fourier series. Australas. Phys. Eng. Sci. Med..

[9] Zhang X., Wang B., Chen X. (2015). Operational Safety Assessment of Turbo Generators with Wavelet Rényi Entropy from Sensor-Dependent Vibration Signals. Sensors.

[10] Xu X., Liang Y., Yang J. (2019). Adaptive Motion Artifact Reduction Based on Empirical Wavelet Transform and Wavelet Thresholding for the Non-Contact ECG Monitoring Systems. Sensors.

[11] Hu H., Zhang L., Yan H., Bai Y., Wang P. (2019). Denoising and Baseline Drift Removal Method of MEMS Hydrophone Signal Based on VMD and Wavelet Threshold Processing. IEEE Access.

[12] Di Biagio V., Cossarini G., Salon S., Lazzari P., Querin S., Sannino G., Solidoro C. (2019). Temporal scales of variability in the Mediterranean Sea ecosystem: Insight from a coupled model. J. Mar. Syst..

[13] Huang N.E., Shen S.S.P. (1998). Hilbert-Huang Transform and Its Applications.

[14] Wu Z., Huang N.E. (2009). Ensemble empirical mode decomposition: A noise-assisted data analysis method. Adv. Adapt. Data Anal..

[15] Yeh J.R., Shieh J.S., Huang N.E. (2010). Complementary Ensemble Empirical Mode Decomposition: A Novel Noise Enhanced Data Analysis Method. Adv. Adapt. Data Anal..

[16] Dragomiretskiy K., Zosso D. (2014). Variational Mode Decomposition. IEEE Trans. Signal Process..

[17] Li Y., Li Y., Chen X., Yu J. (2017). Denoising and Feature Extraction Algorithms Using NPE Combined with VMD and Their Applications in Ship-Radiated Noise. Symmetry.

[18] Li Y., Li Y., Chen X., Yu J. (2018). Research on Ship-Radiated Noise Denoising Using Secondary Variational Mode Decomposition and Correlation Coefficient. Sensors.

[19] Zhang X., Miao Q., Zhang H., Wang L. (2018). A parameter-adaptive VMD method based on grasshopper optimization algorithm to analyze vibration signals from rotating machinery. Mech. Syst. Signal Process..

[20] Miao Y., Zhao M., Lin J. (2019). Identification of mechanical compound-fault based on the improved parameter-adaptive variational mode decomposition. ISA Trans..

[21] Wang Z., Wang J., Du W. (2018). Research on Fault Diagnosis of Gearbox with Improved Variational Mode Decomposition. Sensors.

[22] Li Z., Chen J., Zi Y., Pan J. (2017). Independence-oriented VMD to identify fault feature for wheel set bearing fault diagnosis of high speed locomotive. Mech. Syst. Signal Process..

[23] Yan X., Jia M., Xiang L. (2016). Compound fault diagnosis of rotating machinery based on OVMD and a 1.5-dimension envelope spectrum. Meas. Sci. Technol..

[24] Wang Z., He G., Du W., Zhou J., Han X., Wang J., He H., Guo X., Wang J., Kou Y. (2019). Application of Parameter Optimized Variational Mode Decomposition Method in Fault Diagnosis of Gearbox. IEEE Access.

[25] Vargas R.N., Paschoarelli V., Antônio C. (2018). Electrocardiogram signal denoising by clustering and soft thresholding. IET Signal Process..

[26] Piazzo L., Panuzzo P., Pestalozzi M. (2015). Drift removal by means of alternating least squares with application to Herschel data. Signal Process..

[27] Savitzky A., Golay M. (1964). Smoothing and Differentiation of Data by Simplified Least Squares Procedures. Anal. Chem..

[28] Verma A.K., Saini I., Saini B.S. (2018). The baseline wandering noise removal from ECG signal using forward–backward Riemann Liouville fractional integral-based empirical wavelet transform approach. Int. J. Wavelets Multiresolut. Inf. Process..

[29] Sanyal A., Baral A., Lahiri A. (2012). Application of Framelet Transform in Filtering Baseline Drift from ECG Signals. Procedia Technol..

[30] Anu S., Harjit S., Anureet K. (2018). QRS detection of ECG signal using hybrid derivative and MaMeMi filter by effectively eliminating the baseline wander. Analog Integr. Circ. Signal Process..

[31] Kim J.H., Lee K.H., Lee J.W., Kim K.S. (2018). Semi-real-time removal of baseline fluctuations in electrocardiogram (ECG) signals by an infinite impulse response low-pass filter (IIR-LPF. J. Supercomp..

[32] Yan H., Zhang L. (2019). Denoising of MEMS Vector Hydrophone Signal Based on Empirical Model Wavelet Method. Proceedings.

[33] Ji Y., Wang X., Liu Z., Yan Z., Jiao L., Wang D., Wang J. (2017). EEMD-based online milling chatter detection by fractal dimension and power spectral entropy. Int. J. Adv. Manuf. Technol..

[34] Mirjalili S., Lewis A. (2016). The Whale Optimization Algorithm. Adv. Eng. Softw..

[35] Schevill W.A., Schevill W.E. (1979). Aerial Observation of Feeding Behavior in Four Baleen Whales: *Eubalaena glacialis*, *Balaenoptera borealis*, *Megaptera novaeangliae*, and *Balaenoptera physalus*. J. Mammal..

[36] Kirkpatrick S., Gelatt C.D., Vecchi M.P. (1983). Optimization by simmulated annealing. Science.

[37] Kennedy J., Eberhart R. Particle swarm optimization. Proceedings of the ICNN’95—International Conference on Neural Networks.

[38] Shannon C.E. (1948). A mathematical theory of communication. Bell Labs Tech. J..

[39] Urick R., Kuperman W.A. (1998). Ambient Noise in the Sea. J. Acoust. Soc. Am..

[40] Huang G., Li Z., Wu S., Xue C., Yang S., Zhang W. (2014). A Bionic Fish Cilia Median-Low Frequency Three-Dimensional Piezoresistive MEMS Vector Hydrophone. Nano-Micro Lett..

